# Early Fibroblast Remodeling Associated with Lung Tissue Preservation After FLASH Irradiation

**DOI:** 10.1007/s43657-026-00343-z

**Published:** 2026-08-19

**Authors:** Caihui Li, Luhao Tan, Qixiang Liu, Zhihui Li, Menghua Li, Xuefeng Yang, Guofu Dong, Cheng Quan, Jie Ping, Changzhen Wang, Hao Lu

**Affiliations:** 1 College of Life Sciences, Hebei University, Baoding, 071002 China; 2 Academy of Military Medical Sciences, Beijing, 100850 China; 3 College of Animal Science and Technology, Yangtze University, Jingzhou, 434023 Hubei China

FLASH radiotherapy (FLASH-RT), delivered at ultra-high dose rates, substantially reduces normal tissue toxicity while preserving tumor control (Ma et al. 2024), but its early cellular responses in radiation-induced lung injury (RILI), particularly within stromal fibroblasts, remain poorly understood.

To address this question, we analyzed a murine lung single-cell RNA sequencing (scRNA-seq) dataset collected at one and seven days after FLASH or CONV thoracic irradiation with a 17.8 Gy dose (Lu et al. 2025; Supplementary Information). Mesenchymal analysis identified multiple fibroblast states relatively preserved at day 7 after FLASH versus CONV irradiation (Fig. 1a–c, Fig. S1). Among these, FB_Mdk exhibited distinctive transcriptional features associated with inflammatory modulation, extracellular matrix (ECM) remodeling, fibroblast activation, and metabolism (Fig. 1d, e; Table S1). Trajectory analyses positioned FB_Mdk as an intermediate state (Fig. 1f, Fig. S2), characterized by the strongest communication activity among fibroblast subsets (Fig. 1g), suggesting a prominent role in stromal network regulation. 

**Fig. 1:**
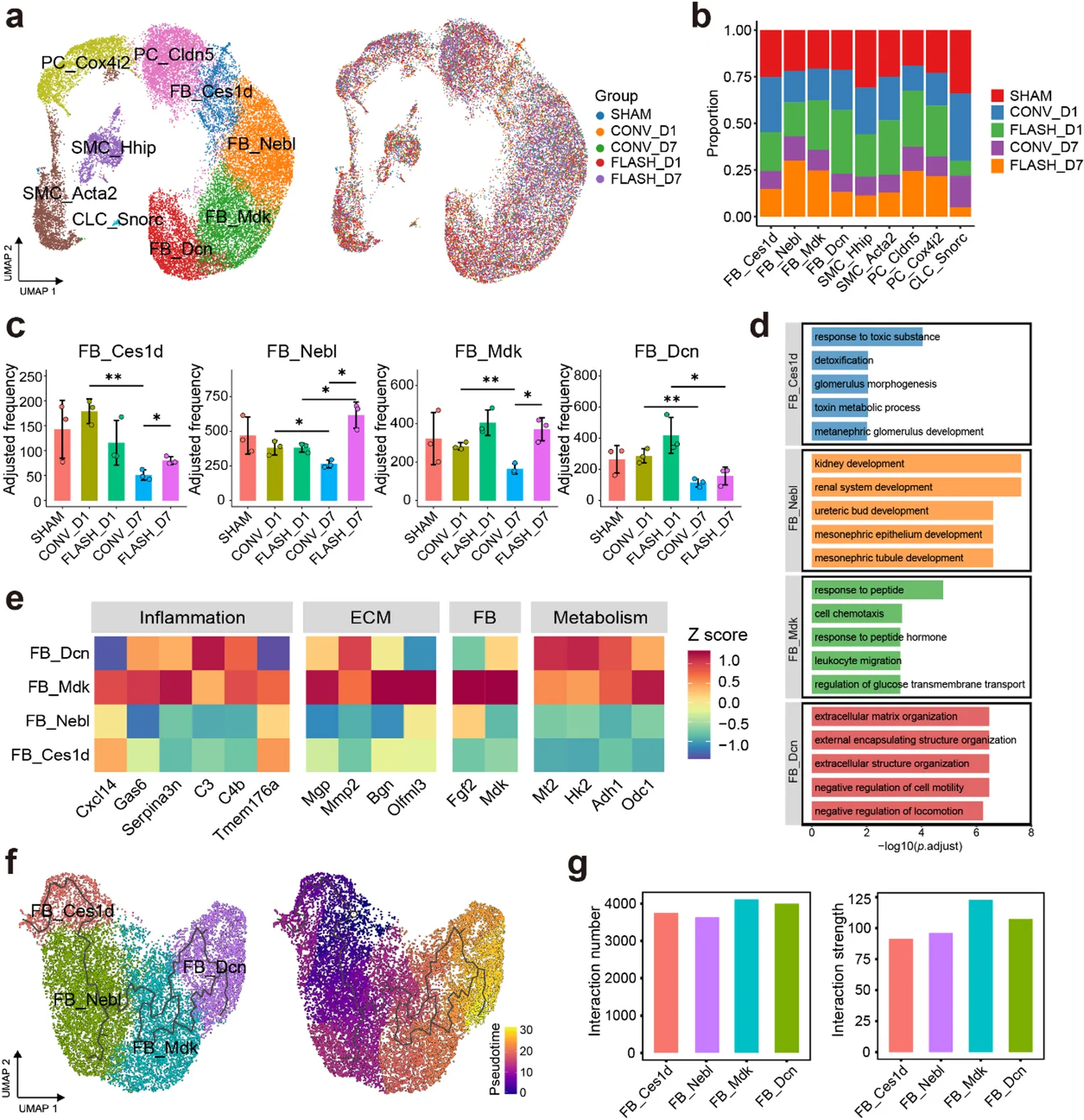
Irradiation-induced mesenchymal remodeling and trajectory dynamics. a Uniform Manifold Approximation and Projection (UMAP) of mesenchymal cells colored by cluster identity (left) and sample (right). b–c Cell proportions (b) and adjusted frequencies (c) of fibroblast subtypes across groups. d Top enriched Gene Ontology (GO) biological processes of fibroblast subtypes. e Functional marker expression across fibroblast clusters. f Trajectory (left) and pseudotime (right) inferred using Monocle v3. g Number (left) and strength (right) of fibroblast interactions in FLASH at seven days post-irradiation (dpi)

Communication pathway analysis further identified the MK signaling pathway, centered on Mdk (midkine), as the dominant pathway associated with FB_Mdk, with greater activity under FLASH than under CONV irradiation (Fig. 2a, b). FB_Mdk preferentially communicated with other mesenchymal populations through Mdk-centered ligand-receptor interactions (Fig. 2c, d, Fig. S3), which have been implicated in matrix remodeling and fibrotic processes (Grivas et al. 2021; Xu et al. 2024). Given the early injury stage analyzed here, these interactions likely reflect adaptive stromal communication rather than established fibrogenic activation. Consistent with these observations, fibroblast transcriptional responses differed markedly between irradiation modalities. FLASH irradiation promoted ECM remodeling and structural organization programs, whereas CONV irradiation induced stress- and inflammation-related signatures (Fig. 2e, Fig. S4; Tables S2–S4). 

**Fig. 2:**
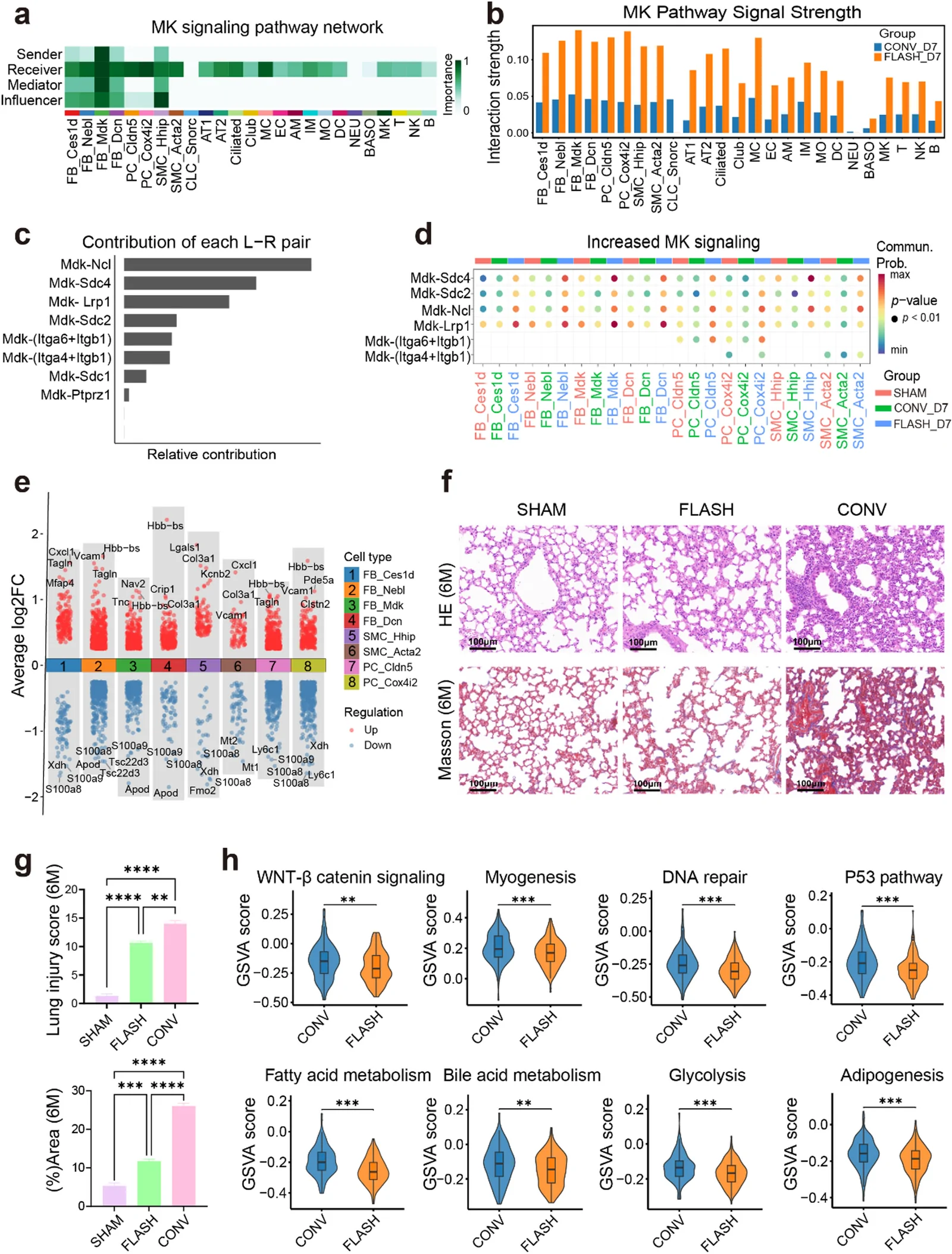
Differential fibroblast responses to FLASH and CONV irradiation. a Heatmap showing cell-type roles in the MK signaling pathway. b MK signaling strength from FB_Mdk to other lung cell types at 7 dpi. c Relative contributions of ligand-receptor pairs to MK signaling. d Ligand-receptor interactions between FB_Mdk and other mesenchymal populations. e Differentially expressed genes (DEGs) between FLASH and CONV at 7 dpi. f–g Representative hematoxylin and eosin (H&E) and Masson’s trichrome staining (f) and Szapiel lung injury and fibrosis scores (g) at 6 months post-irradiation (n = 3; scale bars, 100 μm). h Gene Set Variation Analysis (GSVA) scores of selected pathways in fibroblasts at 5 months post-irradiation (*p < 0.05*, **p < 0.01, ***p < 0.001, ****p < 0.0001)

We next explored whether early fibroblast remodeling was associated with long-term tissue outcomes. Histological analyses at three and six months showed that CONV irradiation caused extensive alveolar disruption and collagen accumulation, whereas FLASH preserved lung architecture and reduced fibrosis (Fig. 2f, g, Fig. S5). Consistently, analysis of an independent late-stage scRNA-seq dataset (Sesink et al. 2024) revealed persistent activation of DNA repair, p53 signaling, and metabolic reprogramming pathways after CONV irradiation, whereas these alterations were attenuated following FLASH irradiation (Fig. 2h). These findings suggest that ECM-associated transcriptional programs in FLASH fibroblasts reflect early coordinated remodeling rather than persistent fibrogenic activation. 

Together, these findings demonstrate that FLASH irradiation preserves multiple fibroblast states and promotes distinct stromal remodeling during early lung injury. Mdk⁺ fibroblasts represent a highly interactive fibroblast state characterized by prominent remodeling-associated transcriptional features and intercellular communication. Although Mdk-mediated signaling has been implicated in chronic fibrosis, its functions are highly context-dependent. The absence of selective fibroblast depletion under FLASH irradiation argues against a model driven by elimination of detrimental fibroblasts. Instead, fibrosis-related outcomes may be shaped by coordinated remodeling across fibroblast states, with Mdk⁺ fibroblasts potentially contributing to adaptive early stromal responses associated with improved tissue preservation.

## Supplementary Information

Below is the link to the electronic supplementary material.

Supplementary Material 1

Supplementary Material 2

## Data Availability

The datasets analyzed in this study are publicly available in the Gene Expression Omnibus (GEO) database (GSE298566 and GSE240510).
