# Supplementary material for: Early Fibroblast Remodeling Associated with Lung Tissue Preservation After FLASH Irradiation
Source: Phenomics. 2026 Aug 19;6(4):530–3. doi: 10.1007/s43657-026-00343-z (PMC13713941; doi:10.1007/s43657-026-00343-z)
Supplement: Supplementary file 1 [file 43657_2026_343_MOESM2_ESM.docx]

**Supplementary Information**

**Early Fibroblast Remodeling Associated with Lung Tissue Preservation after FLASH Irradiation**

## **Methods**

### **Single-cell RNA-sequencing (scRNA-seq) Dataset and Processing**

Single-cell RNA-sequencing (scRNA-seq) data were obtained from our published murine lung dataset (Lu et al. 2025), comprising samples collected at 1 and 7 days post-irradiation (dpi) after 17.8-Gy FLASH or conventional (CONV) thoracic irradiation with a 6-MeV electron beam (mean dose rates: 200 and 0.3 Gy/s, respectively; field size, 2.5 × 6 cm).

Raw FASTQ files were processed using Cell Ranger (10x Genomics, v7.2.0), and filtered matrices were analyzed using Seurat (v4.3.3) (Hao et al. 2021). Cells with <1,000 unique molecular identifier counts, <500 detected genes, or >10% mitochondrial transcripts were excluded. After LogNormalize normalization, 2,000 highly variable genes were selected excluding ribosomal, immunoglobulin, and mitochondrial genes. Data were integrated using Harmony (Korsunsky et al. 2019), followed by clustering, marker identification, and Uniform Manifold Approximation and Projection (UMAP) visualization. Scrublet (Wolock et al. 2019) was used to estimate doublets, and clusters with doublet rates >10% were removed.

### **Intercellular Communication Analysis**

Intercellular signaling networks were inferred from normalized expression data using CellChat (v1.6.1) (Jin et al. 2021; Jin et al. 2025). Ligand-receptor pairs and pathway-level communication probabilities were calculated from normalized expression data and curated interaction priors. Interactions were retained when both interaction partners were expressed in at least 10 cells.

### **Pseudotime Analysis**

Partition-based Graph Abstraction (PAGA) analysis was performed using Scanpy (v1.11.2) (Wolf et al. 2018; Wolf et al. 2019). Force-directed layouts were initialized from PAGA positions. Fibroblast trajectories were inferred using Monocle V3 (Trapnell et al. 2014) and Slingshot (v2.10.0) (Street et al. 2018), with Monocle aligning cells by sample before principal-graph learning and Slingshot using the same UMAP embedding and cluster labels.

### **Protein-Protein Interaction (PPI) Network Analysis**

STRING (Szklarczyk et al. 2025) was used to analyze genes upregulated under FLASH relative to CONV irradiation at 7 dpi and detected in at least five cell types. Medium-confidence interactions were visualized using Cytoscape (v3.9.1).

### **Hematoxylin and Eosin (H&E) Staining and Masson’s Trichrome Staining**

Mice were euthanized by cervical dislocation, and left lungs were paraffin-embedded and sectioned (4 μm) for H&E and Masson’s trichrome staining. Lung morphology was examined by light microscopy (Olympus BX41, Japan). Collagen area fraction (%) was quantified using ImageJ (National Institutes of Health, USA), and pulmonary inflammation was scored using the Szapiel scoring system (Lu et al. 2025).

### **Statistical Analysis**

Validation data are presented as mean ± standard error of the mean (SEM) and analyzed using GraphPad Prism (v9.5.0, GraphPad Software, USA). Data normality was assessed using the Shapiro-Wilk test. Normally distributed data were analyzed using Student’s *t*-test or one-way ANOVA with Tukey’s multiple-comparisons test, whereas non-normally distributed data were analyzed using appropriate non-parametric tests. *P* < 0.05 was considered statistically significant.

## **References**

Lu H, Li M, Quan C et al (2025) FLASH irradiation modulates immune responses and accelerates lung recovery: A single-cell perspective. Adv Sci 12(34):e01797. <https://doi.org/10.1002/advs.202501797>

Hao Y, Hao S, Andersen-Nissen E et al (2021) Integrated analysis of multimodal single-cell data. Cell 184(13):3573-3587.e3529. <https://doi.org/10.1016/j.cell.2021.04.048>

Korsunsky I, Millard N, Fan J et al (2019) Fast, sensitive and accurate integration of single-cell data with Harmony. Nat Methods 16(12):1289-1296. <https://doi.org/10.1038/s41592-019-0619-0>

Wolock SL, Lopez R, Klein AM (2019) Scrublet: computational identification of cell doublets in single-cell transcriptomic data. Cell Syst 8(4):281-291.e289. <https://doi.org/10.1016/j.cels.2018.11.005>

Jin S, Guerrero-Juarez CF, Zhang L et al (2021) Inference and analysis of cell-cell communication using CellChat. Nat Commun 12(1):1088. <https://doi.org/10.1038/s41467-021-21246-9>

Jin S, Plikus MV, Nie Q (2025) CellChat for systematic analysis of cell-cell communication from single-cell transcriptomics. Nat Protoc 20(1):180-219. <https://doi.org/10.1038/s41596-024-01045-4>

Wolf FA, Angerer P, Theis FJ (2018) SCANPY: large-scale single-cell gene expression data analysis. Genome Biol 19(1):15. <https://doi.org/10.1186/s13059-017-1382-0>

Wolf FA, Hamey FK, Plass M et al (2019) PAGA: graph abstraction reconciles clustering with trajectory inference through a topology preserving map of single cells. Genome Biol 20(1):59. <https://doi.org/10.1186/s13059-019-1663-x>

Trapnell C, Cacchiarelli D, Grimsby J et al (2014) The dynamics and regulators of cell fate decisions are revealed by pseudotemporal ordering of single cells. Nat Biotechnol 32(4):381-386. <https://doi.org/10.1038/nbt.2859>

Street K, Risso D, Fletcher RB et al (2018) Slingshot: cell lineage and pseudotime inference for single-cell transcriptomics. BMC Genomics 19(1):477. <https://doi.org/10.1186/s12864-018-4772-0>

Szklarczyk D, Nastou K, Koutrouli M et al (2025) The STRING database in 2025: protein networks with directionality of regulation. Nucleic Acids Res 53(D1):D730-d737. <https://doi.org/10.1093/nar/gkae1113>

Supplementary Figures

**Fig. S1** Identification of cell types in the lung microenvironment at 1 and 7 days after CONV and FLASH irradiation. **a** UMAP projection of 136,058 cells, colored by major lineages (left), annotated cell types (middle), and sample groups (right). **b** Expression of representative marker genes across cell subtypes. **c** Feature plots showing expression of signature genes for each cell type. **d** Adjusted mesenchymal cell frequency normalized to 10,000 cells per sample. (n = 3, **p* < 0.05*, **p* < 0.01). **e** Cell proportions across clusters in each group

**Fig. S2** Trajectory analysis of lung fibroblasts following irradiation. **a** Force-directed graph (left) and differentiation trajectories (right) of mesenchymal clusters with PAGA connectivity. **b** UMAP showing Slingshot-inferred trajectories, with cells colored by cell type (left) or pseudotime (early, red; late, blue) (right)

**Fig. S3** Characteristics of fibroblast populations and their interaction in CONV- and FLASH-induced lung injury. **a** Overall (left) and fibroblast-derived (right) interaction strength across groups. **b** Differences in interaction weights between fibroblast populations and other cell types at 7 dpi. **c** Differences in COLLAGEN (top), LAMININ (middle), and TWEAK (bottom) signaling pathways between mesenchymal populations and other cell types at 7 dpi. Positive values (red) indicate stronger communication in FLASH than CONV, whereas negative values (blue) indicate weaker communication. **d** Top 20 outgoing signaling pathways of the FB_Mdk cluster in the FLASH_D7 group

**Fig. S4** Differential gene expression and pathway analysis in FLASH and CONV irradiation at 7 dpi. **a** PPI networks of genes commonly upregulated in at least five cell types under FLASH versus CONV (top) and CONV versus FLASH (bottom). Node size and color represent node degree. **b** Enriched pathways among genes commonly upregulated under FLASH (red) and CONV (blue)

**Fig. S5** Representative H&E (top) and Masson’s trichrome (bottom) staining of lung sections at 3 months post-irradiation (n = 3, scale bars, 100 μm). Bar charts showing quantification of Szapiel scores (left) and Masson fibrosis scores (right) (**p* < 0.05*, **p* < 0.01*, ***p* <0.001*, ****p* < 0.0001)
